# Immunotherapy With Recombinant Alt a 1 Suppresses Allergic Asthma and Influences T Follicular Cells and Regulatory B Cells in Mice

**DOI:** 10.3389/fimmu.2021.747730

**Published:** 2021-11-05

**Authors:** Juan Liu, Jia Yin

**Affiliations:** ^1^ Department of Allergy, Peking Union Medical College Hospital, Chinese Academy of Medical Sciences, Peking Union Medical College, Beijing, China; ^2^ Department of Allergy, Peking Union Medical College Hospital, Beijing Key Laboratory of Precision Medicine For Diagnosis and Treatment on Allergic Diseases, Beijing, China; ^3^ Department of Allergy, Peking Union Medical College Hospital, National Clinical Research Center for Dermatologic and Immunologic Disease, Beijing, China

**Keywords:** *Alternaria*, recombinant Alt a 1, allergen-specific immunotherapy, allergic asthma, T follicular cells, regulatory B cells

## Abstract

**Background:**

*Alternaria* is a major source of asthma-inducing allergens. Allergen-specific immunotherapy improves the progression of allergic asthma. The current treatment is based on crude Alternaria extracts. Alt a 1 is the predominant allergen in *Alternaria*. However, the treatment efficacy of recombinant Alt a 1 (rAlt a 1) in an asthmatic animal model and its influence on Tfh and Breg cells are unknown.

**Objective:**

To explore the therapeutic treatment effects of rAlt a 1 on the progress of an asthmatic mouse model and its effect on Tfh and Breg cells.

**Methods:**

We synthesized and purified rAlt a 1. *Alternaria*-sensitized and challenged mice received subcutaneous immunotherapy (SCIT) with four different rAlt a 1 dosages (5, 50, 100, and 150 µg) or PBS only. Finally, lung and airway inflammation, mouse mast cell protease 1 (MMCP-1), serum immunoglobulin responses, Tfh and Breg cell levels, and the correlation between asthmatic features (inflammation grades and IL-4 and IL-10 levels) and these two cell types were measured after *Alternaria* rechallenge.

**Results:**

High purity and allergenic potency of rAlt a 1 protein were obtained. Following treatment with four different rAlt a 1 dosages, both lung and airway inflammation ameliorated, including lung pathology, serum MMCP-1 levels, inflammatory cell numbers, and cytokine levels in bronchoalveolar lavage fluid (BALF). Additionally, rAlt a 1-SCIT increased the expression of *Alternaria*-sIgG1, rAlt a 1-sIgG1, rAlt a 1-sIgG2a, and rAlt a 1-sIgG2b in serum. Moreover, the number and percentage of CXCR5^+^PD-1^+^Tfh cells were increased in the PC control, while they decreased in the rAlt a 1-SCIT groups. Meanwhile, the absolute numbers and proportions of Breg cells were evaluated after administration of rAlt a 1. A positive correlation was observed between CXCR5^+^PD-1^+^Tfh cells and inflammation grades (*r* = 0.50, *p* = 0.01), as well as a slightly strong positive relationship with IL-4 (*r* = 0.55, *p* = 0.005) and IL-10 (*r* = 0.58, *p* = 0.003) levels; Breg cells showed an opposite correlation with the grades of inflammation (*r* = -0.68, *p* = 0.0003), along with a negative correlation to IL-4 (*r* = -0.61, *p* = 0.001) and IL-10 (*r* = -0.53, *p* = 0.008) levels.

**Conclusions:**

We verified that treatment with rAlt a 1 can alleviate asthma progression and further have a regulatory effect on Tfh and Breg cells in an *Alternaria*-induced asthmatic mouse model.

## Introduction

*Alternaria alternata* is considered the most common and abundant species that causes allergic asthma, especially in children, and is associated with its severity and persistence (1, 2). Surveys conducted by researchers worldwide indicate that in a skin prick test (SPT) of patients with mold allergy, >70% (3) show a positive reaction for *Alternaria*, and 4.6% (4) of patients with allergic rhinitis in the central city of China are sensitized to *Alternaria*. Although *A. alternata* predominates in outdoor environments, its spores are ubiquitous in the indoor atmosphere and are principally related to sensitization (5).

Alt a 1 is the major allergen produced by *A. alternata* and is mainly located in the spore wall (6). Its protein structures are characterized by a heat-stable, 30-kDa homodimer, and β-barrel with unknown biological functions (7). Some clinical studies (8–11), reveal whether the nature-purified or rAlt a 1 form is sufficient for the diagnosis of *Alternaria* sensitization, given its importance in identifying more than 90% of *Alternaria*-sensitized patients.

Allergen-specific immunotherapy (AIT) is a disease-modifying, antigen-specific, and long-lasting therapy for allergic diseases (12). Conventional AIT for mold routinely employs a whole *Alternaria* extract for SCIT, although several clinical trials have confirmed the treatment efficacy of AIT for natural *Alternaria* extracts in patients with allergic asthma (8, 9). Because its difficulty for obtaining and its adverse effects, including anaphylaxis and poor compliance for patients, exist (13), an innovative and effective therapeutic approach is warranted. Recombinant allergens can be produced with a consistent quality and can also preserve IgE reactivity and T-cell epitopes, which can eliminate some drawbacks of natural extracts (14). Hence, we hypothesized that treatment with rAlt a 1 is an effective and curative method for *Alternaria*-induced allergic asthma.

To date, T follicular cells (Tfh) and regulatory B cells (Breg) have been investigated in allergic diseases (15, 16), and several studies have also reported their role in AIT (17). Tfh cells, a distinct subset of CD4^+^ T-cells, are associated with high-affinity IgE production and isotype switch, which can be found in the secondary lymphoid organs (SLOs), such as the spleen and lymph nodes (LN) (18). Moreover, Tfh cells express C–X–C chemokine receptor type 5 (CXCR5) and programmed cell death protein 1 (PD-1). Studies using mouse models or patient samples have demonstrated that Tfh cells and their subset cell types (Tfh2) contribute to the production of IgE in allergic diseases, attenuate its expression, and induce follicular regulatory T-cells (Tfr) (19). Breg cells produce cytokines IL-10 and IL-35 to regulate the mechanisms of allergic disorders (20); they also express CD24, CD27, CD38, CD1d, and CD5 (21) on their surface. They have the ability to suppress IgE-mediated allergic inflammation and exhibit a low level of circulating phospholipase A_2_-specific Breg cells in patients allergic to venom (22). After accepting *Lolium perenne* peptide (LPP) AIT, an induction of Breg cells was observed, which was related to the generation of allergen-neutralizing IgG4 antibodies (23).

In this study, we aimed to investigate whether immunotherapy with rAlt a 1 can suppress inflammatory responses in a mouse model of *Alternaria*-induced allergic asthma at first then study its effect on the percentages of Tfh and Breg cells. We produced rAlt a 1; its purity and allergenic potency were tested. Furthermore, asthmatic mice were subcutaneously administered four doses of rAlt a 1. The immune response index, Tfh cells, Breg cells, and correlations between different inflammation indicators and these two cell types were estimated. We found that AIT with rAlt a 1 alleviated lung and airway immune responses and decreased the percentage of Tfh cells, whereas that of Breg cells increased after AIT, and their total contrary variations were closely related to inflammation factors (inflammation grades and levels of the cytokines IL-4 and IL-10).

## Materials and Methods

### Vector Construction and Transformation of *Escherichia coli*

A harboring vector for expression in *E. coli* containing Alt a 1 cDNA was synthesized commercially (pCZN1, Biotyscience, Beijing, China). Briefly, the *Alt a 1* gene (GenBank accession no.: P79085) was designed using the PAS (PCR-based accurate synthesis) method that has a 6× protective poly-histidine tag at both ends, and the *Alt a 1* gene was inserted between the NdeI and XbaI sites of the pCZN1 vector. The pCZN1-*Alt a 1* vector plasmid was then transformed into *E. coli* Top 10 (Biotyscience, Beijing, China) *via* heat shock for 90 s, incubated on ice quickly, and plated on Luria–Bertani (LB) medium containing ampicillin according to the manufacturer’s protocols (Biotyscience, Beijing, China).

### Expression and Identification of rAlt a 1 Protein

The pCZN1-*Alt a 1* vector fusion protein was expressed in *E. coli* Top 10 and induced by isopropyl-beta-thiogalactoside according to the manufacturer’s instructions (IPTG, Sigma-Aldrich, St. Louis, MO, USA). In brief, yeast was grown in 800 ml yeast extract peptone dextrose broth at 37°C with shaking at 220 rpm overnight, and 50 µg/ml ampicillin was then added to the media until the optical density (OD_600_) reached 0.6–0.8. Then, 0.5 mM of IPTG was added to induce fusion protein expression in LB media after 4 h of shaking at 37°C. Cells and supernatant in culture media were separated by centrifugation at 10,000 *×g* for 10 min, identified by 12% sodium dodecyl sulfate polyacrylamide (SDS-PAGE), and stained using Coomassie brilliant blue (Beyotime, Beijing, China). The rAlt a 1 protein was a heterodimer, and the cells were cracked using ultrasound and lysis buffer (20 mM Tris–HCl, 1 mM PMSF, bacteria protease inhibitor cocktail, pH 8.0) to denature the inclusion body once the presence of the rAlt a 1 protein has been confirmed. After the cells were washed with washing buffer (20 mM Tris, 1 mM EDTA, 2 M urea, 1 M NaCl, 1% Triton X-100, pH 8.0) and dissolved in lysis buffer (20 mM Tris, 5 mM dithiothreitol, 8 M urea, pH 8.0), the supernatant was collected and dialyzed using binding buffer (20 mM Tris, 0.15 M NaCl, 20 mM imidazole).

### Purification, Electrophoresis, and Immunoblotting Analysis

The supernatant was filtered through a 0.22-μm membrane (Millipore, Burlington, MA, USA) and applied to a Ni-IDA resin that was washed and equilibrated with 20 column volumes in a binding buffer at a flow rate of 1.5 ml/min. Ni-IDA elution buffer (20 mM Tris, 0.15 M NaCl, 500 mM imidazole) was then passed through the column with linear imidazole concentrations (0–50%) for 30 min. rAlt a 1 protein was eluted at a 200 mM imidazole concentration.

Purity was assessed by 12% SDS-PAGE (3 μg/slot) under reducing and non-reducing conditions. The concentration of rAlt a 1 was 1.65 mg/ml, as detected using the BCA Protein Assay Kit (Thermo Fisher Scientific, Waltham, MA, USA). For immunoblotting studies, the separated proteins were transferred to polyvinylidene fluoride (PVDF, Millipore, Burlington, MA, USA) membranes and blocked with 5% non-fat milk in TBST (Solarbio, Beijing, China) for 2 h at room temperature, followed by incubation at 4°C overnight with human sera (1:20) and anti-His tag antibody (Sangon Biotech, Shanghai, China). After washing with TBST for four times, the blots were incubated with horseradish peroxidase (HRP)-conjugated anti-human IgE (Abcam, Cambridge, MA, USA) and goat anti-mouse IgG antibody (Sangon Biotech, Shanghai, China) for 1 h. Finally, the blots were detected by enzyme-linked chemiluminescence.

### Endotoxin Removal and Quantification of rAlt a 1

Potential endotoxins in the rAlt a 1 protein were removed because they are typically produced in *E. coli*. For this purpose, endotoxin removal columns (GenScript, Nanjing, China) were reused three times. First, the protein was replaced with phosphate-buffered saline (PBS) containing 0.5 M NaCl. Second, the resin was regenerated with 5 ml regeneration buffer three times, followed by 6 ml equilibration buffer three times. Third, 6 ml of the protein was loaded onto the column and collected into endotoxin-free tubes.

Endotoxin levels were quantified using a Chromogenic Endotoxin Quant Kit (Thermo Fisher Scientific, Waltham, MA, USA), according to the manufacturer’s protocol. Briefly, 50 µl of protein was added in duplicate in a 96-well plate at 37°C, and the OD was measured at 405 nm immediately after assay completion. The endotoxin concentration of each sample was determined using a high standard curve (0.1–1.0 EU/ml). Purified rAlt a 1 contained minimal endotoxin activity (<1.0 EU/ml).

### Experimental Animals and Grouping

Six- to eight-week-old female BALB/c mice were purchased from Charles River Laboratories (Beijing, China) and housed in a specific pathogen-free (SPF) environment at the Laboratory Animal Center of Peking Union Medical University for 1 week prior to the experiments. All protocols were approved by the Institutional Animal Care and Use Committee of the Peking Union Medical University (XHDW-2020-046). A total of 36 mice were divided into the following six groups: negative control group (NC), positive control group (PC), and rAlt a 1 SCIT groups (5, 50, 100, and 150 μg).

### Allergic Asthma Treatment Protocol

Mice in the PC, 5-, 50-, 100-, and 150-μg SCIT groups were sensitized by intraperitoneal injection of 10 μg A. *alternata* (Greer, Lenoir, NC, USA) adsorbed to 1 mg alum (Imject, Pierce) in 100 μl PBS. Challenges were performed by intranasal instillation of 25 μg *A. alternata* in 50 μl PBS under isoflurane anesthesia. SCIT was administered by seven subcutaneous injections on the neck skin on alternate days using PBS for the PC group or 5, 50, 100, and 150 μg purified rAlt a 1 in 100 µl PBS for the rAlt a 1-SCIT groups. The NC group mice were administered with PBS only at all times (**Figure 2A** and **Supplementary Table S1**).

### Assessment of Airway Inflammation

Twenty-four hours after the final challenge, the mice were euthanized with sodium pentobarbital overdose. BALF was collected by flushing the lungs three times with 0.5 ml of PBS through an intravenous catheter and centrifuged at 1,500 rpm for 10 min at 4°C. Levels of IL-13 (Cusabio, Wuhan, China), IL-10, and IL-4 in BALF supernatant were detected by ELISA (Mabtech AB, Nacka Strand, Sweden). BALF total cellular counts were calculated using Cellometer Mini Automated Cell Counter (Nexcelom Bioscience, USA). The differential cell counts were processed by Giemsa staining (Solarbio, Beijing, China). At least 200 cells were counted for each slide by using the microscope (Nikon, Japan).

### Histological Analysis

Whole lung tissues were fixed in 10% neutral-buffered formalin (Biomics Biotech, Nantong, China) for histological analysis. Paraffin-embedded lung tissues (5 μm thick) were subjected to hematoxylin and eosin (H&E) and periodic acid–Schiff (PAS) staining to evaluate tissue inflammation and goblet cell metaplasia. Histological scores were determined by randomly selecting 10 different fields under a microscope according to Liu’s method (24) and calculating the number of inflammatory cells directly. The mucus hypersecretion score by PAS staining was determined as follows: the percentage of the mucus-positive area of the whole bronchus was ≤5% = 0, 5%–25% = 1, 25%–50% = 2, 50%–75% = 3, and >75% = 4. All scores were evaluated by a blinded pathologist (25).

### MMCP-1, Ag-Specific IgE, IgG1, IgG2a, and sIgG2b Detection

Blood was collected at the end of the experiment, and serum was acquired after allowing blood to stand at room temperature for at least 2 h. Serum MMCP-1 levels were measured using a commercially available Deluxe Set ELISA kit (BioLegend, San Diego, CA, USA). Total IgE (T-IgE), *A. alternata* and rAlt a 1-sIgE (Mabtech AB, Nacka Strand, Sweden), sIgG1, sIgG2a, and sIgG2b (Southern Biotechnology Inc., AL, USA) in serum were measured by ELISA according to the manufacturers’ instructions. For detecting A. *alternata*-IgE, IgG, and rAlt a 1-IgE, IgG, *Alternaria* extracts (10 μg/ml), and rAlt a 1 (2 μg/ml) were incubated in carbonate buffer in a 96-well plate.

### Flow Cytometry

Single-cell suspensions of the spleen were harvested and incubated with live/dead fixable dye to exclude dead cells and stained with anti-mouse fluorochrome-conjugated mAbs against CD4 (FITC, GK1.5), CXCR5 (APC-Cy7, L138D7), PD-1 (PE-Cy7, 29F. 1A12), ICOS (PerCP-Cy5.5, 7E.17G9), CD19 (FITC, ID3/CD19), CD5 (PE, 53-7.3), and CD1d (BV421, 1B1) at 4°C in the dark for 30 min. All antibodies were purchased from BioLegend (San Diego, CA, USA). Flow cytometry was performed on LSRII (BD Biosciences, San Diego, CA, USA), and all data were analyzed using FlowJo Software.

### Statistical Analysis

Data are presented as the means ± S.E.M.; all data were assessed for its distribution and homogeneity of variance at first. The Mann–Whitney *U* test was used to compare the significant difference between the two groups. Spearman’s correlation analysis was used to determine the statistical relevance. Statistical significance was set at *p* < 0.05. All statistical analyses were performed using GraphPad Prism Software 9.0 (San Diego, CA, USA).

## Results

### The Synthesized rAlt a 1 Displays a High Purity and Allergenic Potency

**Figure 1:**
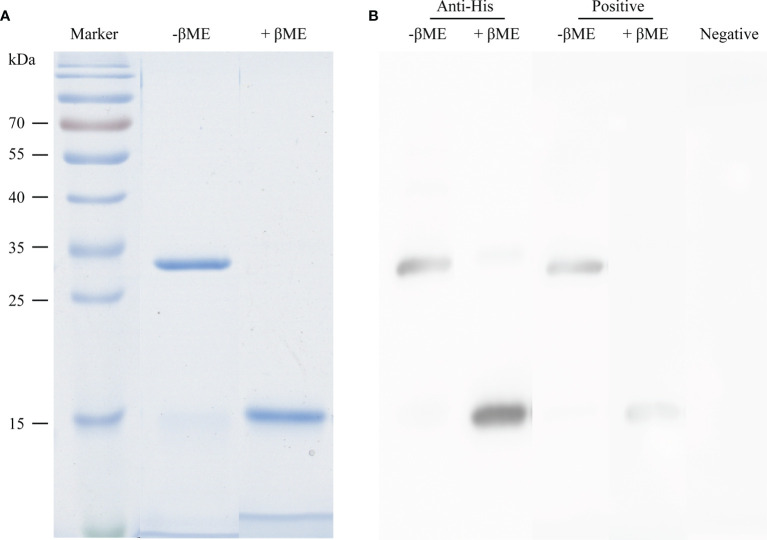
High purity and allergenic potency of rAlt a 1 in the presence and absence of β-mercaptoethanol (βME). **(A)** SDS-PAGE and Coomassie staining of rAlt a 1. **(B)** Western blotting using the anti-His tag antibody and serum from *Alternaria*-allergic and non-allergic patients.

To evaluate the therapeutic effect of rAlt a 1 in SCIT, we established a murine model of *Alternaria*-induced allergy. First, we synthesized, purified, and produced the rAlt a 1 protein. It was a 30-kDa heterodimer made up of 15.3- and 16.4-kDa subunits, as expected (26). SDS-PAGE analysis confirmed that rAlt a 1 showed high purity (>98%) both under non-reducing and reducing conditions (**Figure 1A**). Moreover, to verify the allergenic potency of rAlt a 1, we conducted Western blotting assay using sera from *Alternaria*-allergic and non-allergic patients. We found that rAlt a 1 could bind to IgE epitopes of samples from patients with *Alternaria*-allergy, but no reaction with negative serum (**Figure 1B**).

### rAlt a 1 Suppresses Established Lung Inflammation in Asthmatic Mice

**Figure 2:**
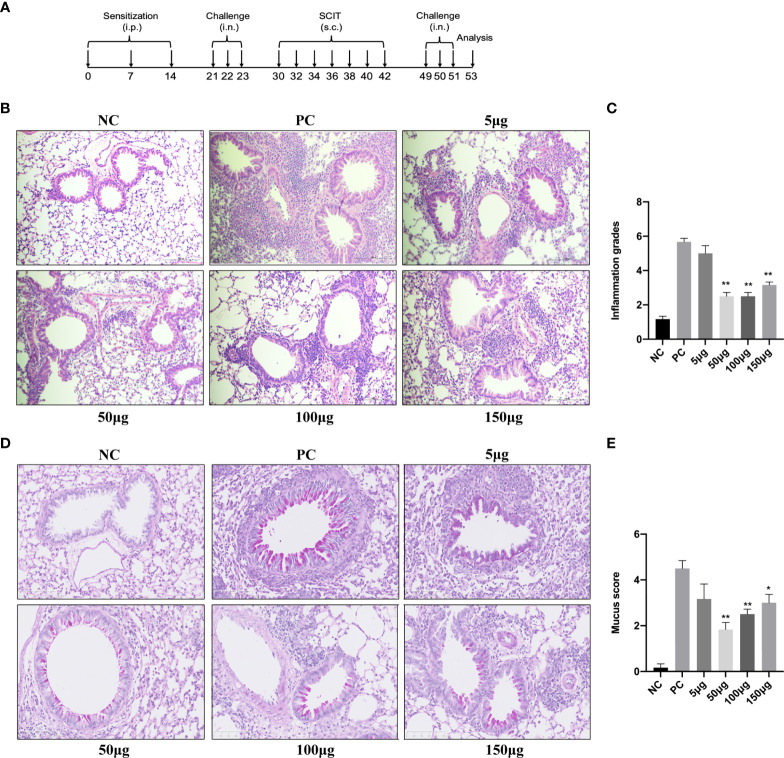
rAlt a 1 suppresses lung inflammation in *Alternar*ia-induced asthmatic mice. **(A)** Schematic diagram of the experimental protocol for asthmatic mice. **(B)** H&E staining of the lung tissues in six groups. *Scale bar* = 100 μm. **(C)** Semi-quantitative analysis of the inflammation scores in lung histology. **(D)** Representative PAS-stained lung histology sections of the NC, PC, and SCIT groups. *Scale bar* = 100 μm. **(E)** Mucus hypersecretion scores of lung tissues in asthmatic mice of each group. (n = 6 per group). Values are means ± SEMs. *p < .05, **p < .01 compared to the PC group. NC, negative group; PC, positive group; 5 μg, 50 μg, 100 μg, 150 μg: 5 μg, 50 μg, 100 μg, 150 μg rAlt a 1 SCIT group. i.p., intraperitoneally; i.n., intranasally; s.c., subcutaneous injection.

Although Alt a 1 has been associated with an increased rate of diagnosing *Alternaria* alternata sensitization (10), its treatment effect remains to be tested in animal models. In our study, an established mouse model that is induced by *Alternaria* and rAlt a 1-SCIT was used to evaluate whether a therapeutic effect for rAlt a 1 was observed (**Figure 2A**). We found that the airways in mice were narrowed and surrounded by numerous inflammatory cells, lung structures were extensively damaged, including goblet cell hyperplasia, and epithelial cells were destroyed. However, the infiltration of lung inflammatory cells and goblet cells and increased scores were alleviated after rAlt a 1 treatment (**Figures 2B–E**), especially in the 50- and 100-μg SCIT groups. Taken together, our data indicated that rAlt a 1 could suppress the allergic immune response with optimal dosages of 50 and 100 μg.

### rAlt a 1 Alleviates BALF Cell Numbers, Cytokine Levels, and Serum MMCP-1 Levels

**Figure 3:**
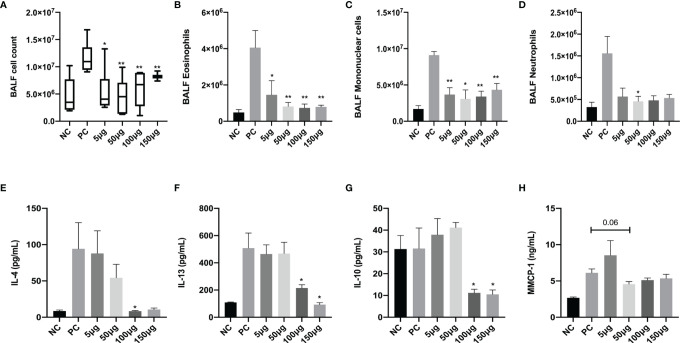
Total inflammatory cells, eosinophils, mononuclear cells, neutrophils, IL-4, IL-13, and IL-10 levels in BALF and MMCP-1 levels in serum decreased after rAlt a 1 treatment. **(A–D)** Total cell counts and the differential cells in BALF. **(E–G)** Quantification of IL-4, IL-13, and IL-10 levels in BALF by ELISA. **(H)** Serum MMCP-1 levels (n = 6 per group). Values are means ± SEMs. *p < .05, **p < .01 compared to the PC group. NC, negative group; PC, positive group; 5 μg, 50 μg, 100 μg, 150 μg: 5 μg, 50 μg, 100 μg, 150 μg rAlt a 1 SCIT group.

We questioned whether successful immunotherapy with rAlt a 1 is effective not only in downregulating airway inflammation and related cytokine response after challenge but also in reducing *Alternaria*-induced anaphylaxis in our mouse model. Therefore, we assessed the total number of inflammatory cells, eosinophils, neutrophils, mononuclear cells, and IL-4, IL-13, and IL-10 levels in BALF at first (**Figure 3**). Remarkably, *Alternaria* challenges in the PC group induced a pronounced increase in the inflammatory cells and IL-4 and IL-13 levels compared to the NC group (*p* < 0.05), although there were comparable IL-10 levels between the NC and PC groups. After administrating different dosages of rAlt a 1, the total and differential cell numbers and IL-13 and IL-4 levels decreased in four dosages of rAlt a 1 groups (**Figures 3A–F**), and even though IL-10 levels had a slight increase in the 5- and 50-μg SCIT groups, the decrease in the 100- and 150-μg SCIT groups was significant (**Figure 3G**). As a marker of mast cell degranulation, serum MMCP-1 level also showed a clear tendency to be lower in rAlt a 1-SCIT groups than in the PC group, although the difference was not significant (**Figure 3H**).

### rAlt a 1 SCIT Influences Serum Immunoglobulin Responses

**Figure 4:**
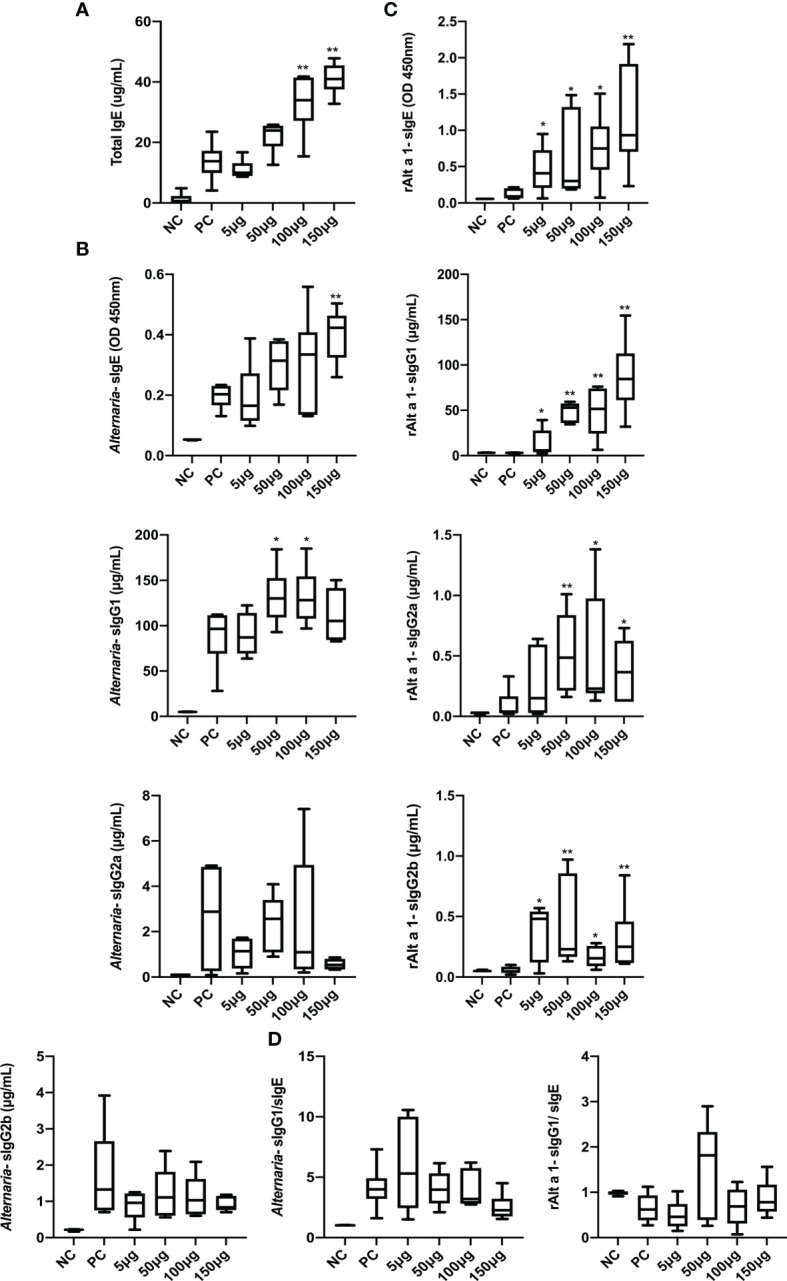
The responses of serum immunoglobulin after rAlt a 1 treatment. **(A)** T-IgE levels in serum after *Alternaria* challenges. **(B)** Serum *Alternaria*-sIgE, *Alternaria*-sIgG1, *Alternaria*-sIgG2a, and *Alternaria*-sIgG2b levels. **(C)** The expressions of rAlt a 1-sIgE, rAlt a 1-sIgG1, rAlt a 1-sIgG2a, and rAlt a 1-sIgG2b. **(D)** The levels of neutralizing antibodies in serum (n = 6 per group). Values are means ± SEMs. *p < .05, **p < .01 compared to the PC group. NC, negative group; PC, positive group; 5 μg, 50 μg, 100 μg, 150 μg: 5 μg, 50 μg, 100 μg, 150 μg rAlt a 1 SCIT group.

To test whether rAlt a 1-SCIT affected *Alternaria*-specific serum immunoglobulin responses, we measured levels of T-IgE (**Figure 4A**), *Alternaria*-sIgE, *Alternaria*-sIgG1, *Alternaria*-sIgG2a, and *Alternaria*-sIgG2b (**Figure 4B**) in mouse serum at the end of the study. Interestingly, administration of rAlt a 1-SCIT induced the levels of T-IgE and sIgE, in a dose-dependent manner. In contrast, the expression of *Alternaria*-sIgG1 significantly improved in the 50- and 100-μg SCIT groups compared to the PC group. However, we observed that SCIT with rAlt a 1 had no remarkable influence on the levels of *Alternaria*-sIgG2a and *Alternaria*-sIgG2b.

We further studied the efficacy and role of rAlt a 1-SCIT on the expression of rAlt a 1-related immunoglobulins, namely, rAlt a 1-sIgE, rAlt a 1-sIgG1, rAlt a 1-sIgG2a, and rAlt a 1-sIgG2b (**Figure 4C**). Importantly, after the *Alternaria* challenge, levels of these immunoglobulins were significantly higher in the 50- and 100-μg SCIT groups than in the PC group. In addition, we measured the capacity of neutralizing antibodies after rAlt a 1-SCIT and found that the ratio of *Alternaria*-sIgG1/*Alternaria*-sIgE levels and that of rAlt a 1-sIgG1/rAlt a 1-sIgE levels did not increase compared to the PC group (**Figure 4D**).

Taken together, these results demonstrated that rAlt a 1-SCIT induced strong *Alternaria*-sIgG1, rAlt a 1-sIgG1, rAlt a 1-sIgG2a, and rAlt a 1-sIgG2b responses, with an increase in IgE levels in the SCIT groups, while the levels of neutralizing antibodies were not altered.

### rAlt a 1 SCIT Decreases the Expression of Tfh Cells and Increases That of Breg Cells

**Figure 5:**
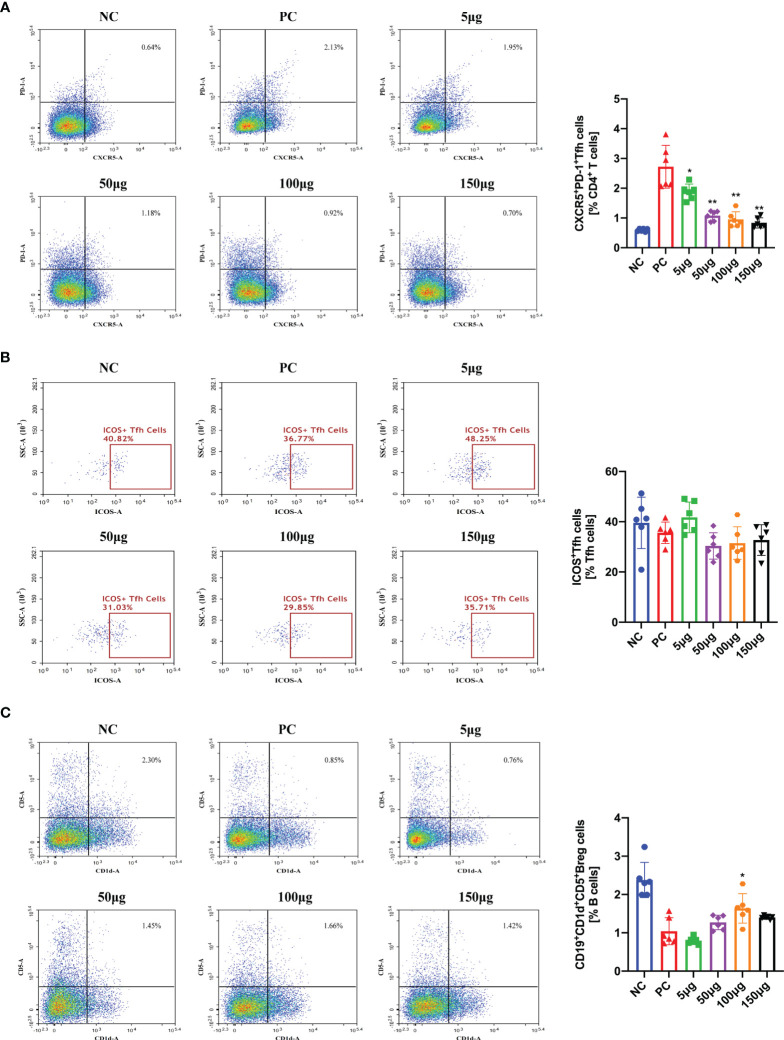
The percentages of Tfh cells were decreased while Breg cells were evaluated following rAlt a 1 subcutaneous treatment. **(A)** Flow cytometry analysis and quantification of Tfh cells in spleen tissues. **(B)** The percentages of ICOS^+^Tfh cells in spleen by using flow cytometry. **(C)** The proportions of Breg cells in spleen and its quantification. *Symbols* represent individual samples (n = 6 per group). Values are means ± SEMs. *p < .05, **p < .01 compared to the PC group. NC, negative group; PC, positive group; 5 μg, 50 μg, 100 μg, 150 μg: 5 μg, 50 μg, 100 μg, 150 μg rAlt a 1 SCIT group.

Recently, increasing evidence has shown that Tfh cells play a key role in the pathology of asthma (15) and AIT in human (27). In our study, the numbers and percentages of Tfh, which is defined as CD4^+^CXCR5^+^PD-1^+^ cells (**Supplementary Figure S1**), in the PC group were significantly increased. Conversely, following rAlt a 1 SCIT, there was a remarkable improvement in Tfh cells in a dose-dependent manner (**Supplementary Figure S2A** and **Figure 5A**). We also investigated the expression of ICOS^+^Tfh cells (in total Tfh cells) after rAlt a 1 SCIT because ICOS maintains the signaling of Tfh cells (28); however, there was no significant difference in the percentage of ICOS^+^Tfh cells between the PC and rAlt a 1 SCIT groups (**Figure 5B**). In addition, we further assessed Breg cells (CD19^+^CD1d^+^CD5^+^) as a negative regulator of immunity (29) in asthma and SCIT groups. Notably, the proportion and numbers of Breg cells in the PC group predominantly decreased, and increased following rAlt a 1 SCIT, although only the 100-µg SCIT group showed statistical significance (**Figure 5C**, **Supplementary Figure S2B**). Taken together, our results suggest that the balance of Tfh and Breg cells in an asthmatic mouse model could be modified by rAlt a 1 SCIT.

### The Correlation Between Asthmatic Features and Tfh and Breg Cells After rAlt a 1 SCIT

**Figure 6:**
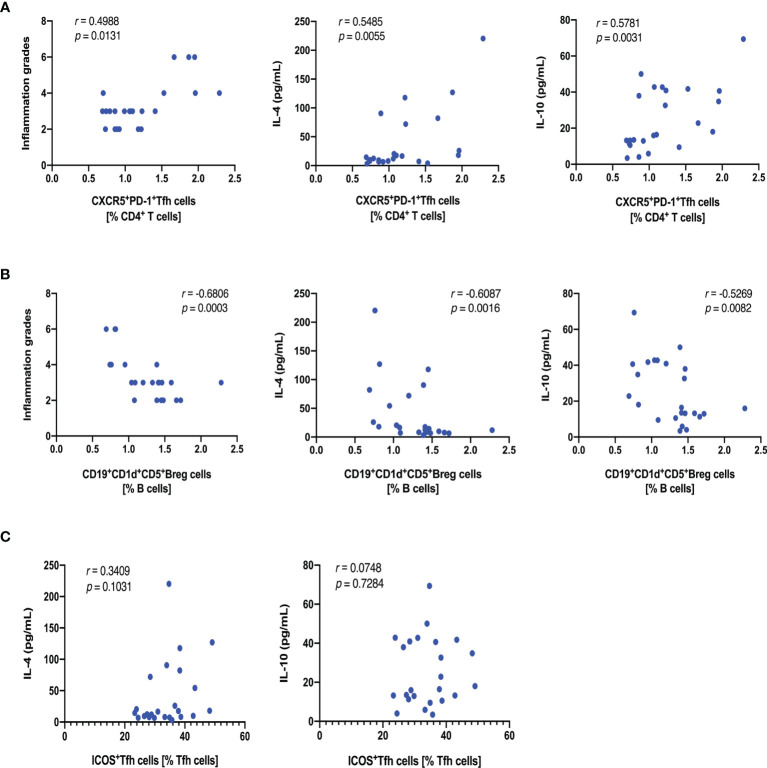
Correlations between asthmatic feathers and Tfh and Breg cells after rAlt a 1 SCIT. **(A)** Scatter diagram showing Tfh cells *vs*. inflammation grades, IL-4, and IL-10 levels (n = 24). **(B)** Graph showing Breg cells *vs*. inflammation grades, IL-4 and IL-10 levels (n = 24). **(C)** Graph showing ICOS^+^Tfh cells *vs*. IL-4 and IL-10 levels (n = 24). *Symbols* represent individual samples.

We hypothesized that a relative decrease in Tfh cells and an increase in Breg cells play crucial roles in the deterioration of asthma; in contrast, rAlt a 1 SCIT changed this balance. As a result, a positive correlation was observed between the frequencies of Tfh cells and inflammation grades and IL-4 and IL-10 levels (**Figure 6A**) in our study. However, the proportion of Breg cells was negatively correlated with the inflammation grades in lung tissues and IL-4 and IL-10 levels in BALF (**Figure 6B**). In addition, such correlations were not observed between the percentages of ICOS^+^Tfh cells and IL-10 levels, even though a positive relationship was observed between the ICOS^+^Tfh cells and IL-4 levels (**Figure 6C**).

## Discussion

To date, there are only a few studies of AIT for fungi, especially for *Alternaria* spp.; a majority of them mainly focus on crude *Alternaria* extracts (30), with only two clinical trials with purified natural Alt a 1 (nAlt a 1) (8, 9) and one with B-cell epitopes in mice (31). Alt a 1 is the most relevant allergen with a prevalence of >90% in patients sensitized to *Alternaria.* Clinical studies have already confirmed that AIT for nAlt a 1 is associated with a great improvement in combined symptom and medication score (CSMS) and upregulated IgG4 levels (8), which suggests that Alt a 1 provides a positive feedback in immunotherapy in terms of efficacy and immune response.

Advantages of rAlt a 1 include high quality of vaccines and proportion of epitopes, which preserves the reactivity of IgE and induces allergen-specific blocking IgGs (14). Our immunoblotting results of rAlt a 1 displayed the same characteristics. More importantly, purified Alt a 1 excludes potential complex proteins, including mycotoxins, chitin, mannans, β-1,3-glucans, and endotoxins, which possibly activate a proinflammatory innate response (32). In addition, studies have demonstrated the efficacy of native-like recombinant allergens equal to allergen extracts and purified natural allergens, although they have the same side effects as natural allergens (33, 34).

Accumulating evidence suggests that the migration of inflammatory cells to the airways and lung tissues is caused by innate and adaptive immune responses in allergic asthma. In our study, pathological histology in the PC group showed numerous inflammatory cells surrounded, goblet cells, and alveolar septum damaged. However, rAlt a 1-SCIT changed the pathophysiology to a large extent and decreased cytokines and total and differential inflammatory cell numbers in BALF. Studies have highlighted the role of IL-10 in the immune regulation of allergic diseases and AIT. Elevated IL-10 levels were found after immunotherapy for bee-venom allergies (22). IL-10 mRNA levels in serum correlated with AIT efficacy in house dust mite (HDM)-induced asthma in humans (35). Notably, IL-10 levels in BALF increased in the 5- and 50-μg SCIT groups but not in the 100 and 150 μg-SCIT groups, possibly because the cytokines in BALF did not reflect the real levels of IL-10 in the serum, and cytokine levels after AIT were influenced by the circadian clock. A previous study on grass pollen sublingual immunotherapy (SLIT) concluded that the gene expression and secretion of IL-10 were downregulated (33), which is in line with our results. Meanwhile, a Japanese study reported that mice that received SLIT at 10 a.m. (resting phase) and 10 p.m. (active phase) presented differences in the reduction levels of IL-4, IL-10, and IL-13. Altogether, the experimental mouse model of rAlt a 1-SCIT treatment generally ameliorated Th2 inflammation.

MMCP-1 in serum acts as an indicator of anaphylaxis. In this model, we did not investigate in depth the relative hypothermia like food allergen models, but the decreased tendency of MMCP-1 in SCIT groups indicated that Alt a 1 recognized IgE-mediated mast cell degranulation, rather than an alternative pathway consisted by IgG1-antigen immune complex (36). The degree of anaphylaxis is mild but not severe, which may explain this statistical analysis.

In clinical studies of AIT, local immunoglobulin responses, particularly antigen-specific neutralizing antibodies, are often considered as biomarkers of AIT. However, a review summarized that these tolerance antibodies reflect high levels of allergen exposure only during AIT and correlate poorly with clinical efficacy (37). Interestingly, both T-IgE and *Alternaria*/rAlt a 1-sIgE levels improved with the increase in therapeutic concentrations compared to the PC group in our asthmatic model. Such responses have been investigated in another animal study on *Alternaria* B-cell epitope immunotherapy, where mimotope treatment reduced IgE reactions (31). In contrast, SCIT with purified Der p1/2 in a murine asthma model showed upregulated levels of total and antigen-specific IgE after rechallenge (38). Evidence showed that a reduction of allergen-specific IgE levels could be observed after long subcutaneous immunotherapy over several years (39), and it remained a long half-life once they combined with allergens. Collectively, this paradoxical transient of IgE levels appears to be associated with the method of treatment, the concentration of drugs, and route of delivery (40). Another finding in our model was the induction of blocking IgGs, which has been suggested as a therapeutic mechanism. Notably, we only observed a positive response of *Alternaria*-sIgG1 but levels of all sIgG antibodies against rAlt a 1 increased upon SCIT. The absence of *Alternaria*-sIgG2a, 2b, and the ratio of sIgG1/sIgE could be explained by the numerous non-protein dose of the full extract than that of rAlt a 1, which acts on innate immune cells. In agreement with our results, SCIT with HDM and Der p1/2 at the same time did not induce a strong neutralizing antibody response for HDM (38). However, Siebeneicher et al. (41) indicated that AIT for rBet v1 or its hypoallergenic form (rBet v 1B2) had similar levels of sIgG1 and sIgG2a compared to the PBS group, and they speculated that these antibodies were not a mechanism for the therapeutic efficacy of rBet v 1B2. Overall, our results demonstrated that high levels of serum immunoglobulins occurred after rAlt a 1-SCIT.

More recently, the novel subset of CD4^+^ T-cells (Tfh cells), which was introduced into allergic diseases and AIT, plays a crucial role in the production of IgE and contributes to the class switching of antibodies (42). A previous study has shown that the generation of high-affinity allergen-specific IgE is dependent on Tfh13 (43). In addition, many clinical trials have revealed a lower frequency of circulating Tfh (cTfh) cells in AIT-administered patients than in those not receiving AIT (27, 44, 45). However, cTfh cells are not *bona fide* Tfh despite common characteristics (46). It is, therefore, important to note the peculiar role of Tfh cells in an animal model of asthma after AIT. In our study, we compared the percentages of Tfh and ICOS^+^Tfh cells between the PC and rAlt 1-SCIT groups. Consistent with the expression of cTfh cells in patients with allergy (47) and other animal models (48), an initial upregulation of Tfh cells was observed to decrease sharply in SCIT groups in a dose-dependent manner. In contrast, the number of ICOS^+^Tfh cells was not significant, although ICOS/ICOS-L signaling is essential for the generation of Tfh cells, disrupting this interaction or ICOS deficiency limits the development of Tfh cells either in patients or in mice (49), and blocking ICOS alleviates the inflammation of *Alternaria*- and HDM-induced allergy in mouse models (50). Even though this inconsistency of Tfh and ICOS^+^Tfh cells in our model requires further exploration, our study found that rAlt a 1-SCIT induced an increased proportion of Tfh cells.

In addition to this salient feature of Tfh cells, we showed a decline in the proportion of Breg cells from a normal condition to asthmatic and converted in the rAlt a 1-SCIT groups. Breg cells have a negative regulatory capacity by secreting IL-10 in allergic diseases (15) while acting as a tolerance hallmark following AIT. Studies have indicated that the number of Breg cells and plasmablasts increased in grass pollen- (51) and HDM-AIT (52) patients, which is significantly related to the improvement of clinical symptoms throughout AIT. Collectively, these results suggest that the expression of Breg cells is impaired in allergic diseases but upregulated in AIT.

It is of great interest to know whether AIT affects Tfh or Breg cells in relation to the levels of IL-4 and IL-10 in a murine asthma model. Thus, we analyzed the relationships between inflammation grades and IL-4 and IL-10 levels, and Tfh and Breg cells following AIT. We found that the reduction in the percentage of Tfh cells after AIT was positively related to these inflammatory indicators, whereas Breg cells showed significant negative correlations, which further proved the role of Tfh and Breg cells in the mechanisms of asthma in mice. Additionally, we speculate that Tfh and Breg cells regulate immune balance by secreting IL-4 and IL-10 in asthmatic models, although we did not explore its precise mechanism. To the best of our knowledge, this study is the first to display these correlations in rAlt a 1-SCIT asthmatic models.

The study is not free of limitations. First, the allergenic potency of rAlt a 1 was not investigated using circular dichroism or granulation of basophils and mast cells. Second, we compared the therapeutic efficacy of recombinant Alt a 1 only between rAlt a 1-SCIT and PC groups. A nAlt a 1-SCIT group would have been beneficial in elucidating our observations. Third, airway hyperreactivity (AHR), a hallmark of asthma inflammation, was not examined owing to the lack of a suitable experimental device.

Further analyses of the percentages of Tfh and Breg cells in LN and lung tissues of asthmatic mice as well as the expression of antigen-specific Tfh cells and Breg cells are necessary. In addition, an in-depth understanding of the mechanism underlying the change in these two cell types induced by rAlt a 1 needs to be investigated; we consider that microRNA might mediate the development and function of Tfh cells (53).

In conclusion, our study first confirmed that rAlt a 1 subcutaneous AIT can improve inflammatory responses in an *Alternaria*-induced asthmatic mouse model. Meanwhile, SCIT of rAlt a 1 resulted in the modulation of Tfh and Breg cells, and the underlying changes correlated with inflammation features. All these findings suggest a potential treatment for *Alternaria*-induced asthma.

## Data Availability Statement

The original contributions presented in the study are included in the article/**Supplementary Material**. Further inquiries can be directed to the corresponding author.

## Ethics Statement

The animal study was reviewed and approved by the Institutional Animal Care and Use Committee of Peking Union Medical University.

## Author Contributions

JL performed the experiments, analyzed the data, and drafted this manuscript. JY designed the experiments and provided the funding. All authors contributed to the article and approved the submitted version.

## Funding

This study was supported by CAMS Initiative for Innovative Medicine (CAMS-I2M), Grant/Award Number: 2016-I2M-1-003 and Major National Science and Technology Projects for “Significant New Drugs Creation”, Grant/Award Number: 2019ZX09301131.

## Conflict of Interest

The authors declare that the research was conducted in the absence of any commercial or financial relationships that could be construed as a potential conflict of interest.

## Publisher’s Note

All claims expressed in this article are solely those of the authors and do not necessarily represent those of their affiliated organizations, or those of the publisher, the editors and the reviewers. Any product that may be evaluated in this article, or claim that may be made by its manufacturer, is not guaranteed or endorsed by the publisher.

## Supplementary Material

Supplementary Figure S1 Gating strategy used to define Tfh, ICOS^+^ Tfh, and Breg cells in the spleen of asthmatic mice. Within the live CD4^+^ T cells, Tfh cells were identified as CXCR5^+^PD-1^+^ cells. ICOS^+^ Tfh cells were identified from the Tfh cells as CXCR5^+^PD-1^+^ ICOS^+^ cells. Within the live CD19^+^ B cells, Breg cells were identified as CD5^+^CD1d^+^ cells.

Supplementary Figure S2 Absolute number of Tfh and Breg cells in the spleen of asthmatic mice. **(A)** Tfh cell numbers in each group. **(B)** Breg cell numbers in each group. (n=6 per group). Values are means ± SEMs. **P* < .05, ***P* < .01 compared to the PC group. NC, Negative group; PC, Positive group, 5 μg, 50 μg, 100 μg, 150 μg: 5 μg, 50 μg, 100 μg, 150 μg rAlt a 1 SCIT group.
