# Supplementary figures and images for: Immunotherapy With Recombinant Alt a 1 Suppresses Allergic Asthma and Influences T Follicular Cells and Regulatory B Cells in Mice
Source: Front Immunol. 2021 Nov 5;12:747730. doi: 10.3389/fimmu.2021.747730 (PMC8602824; doi:10.3389/fimmu.2021.747730)

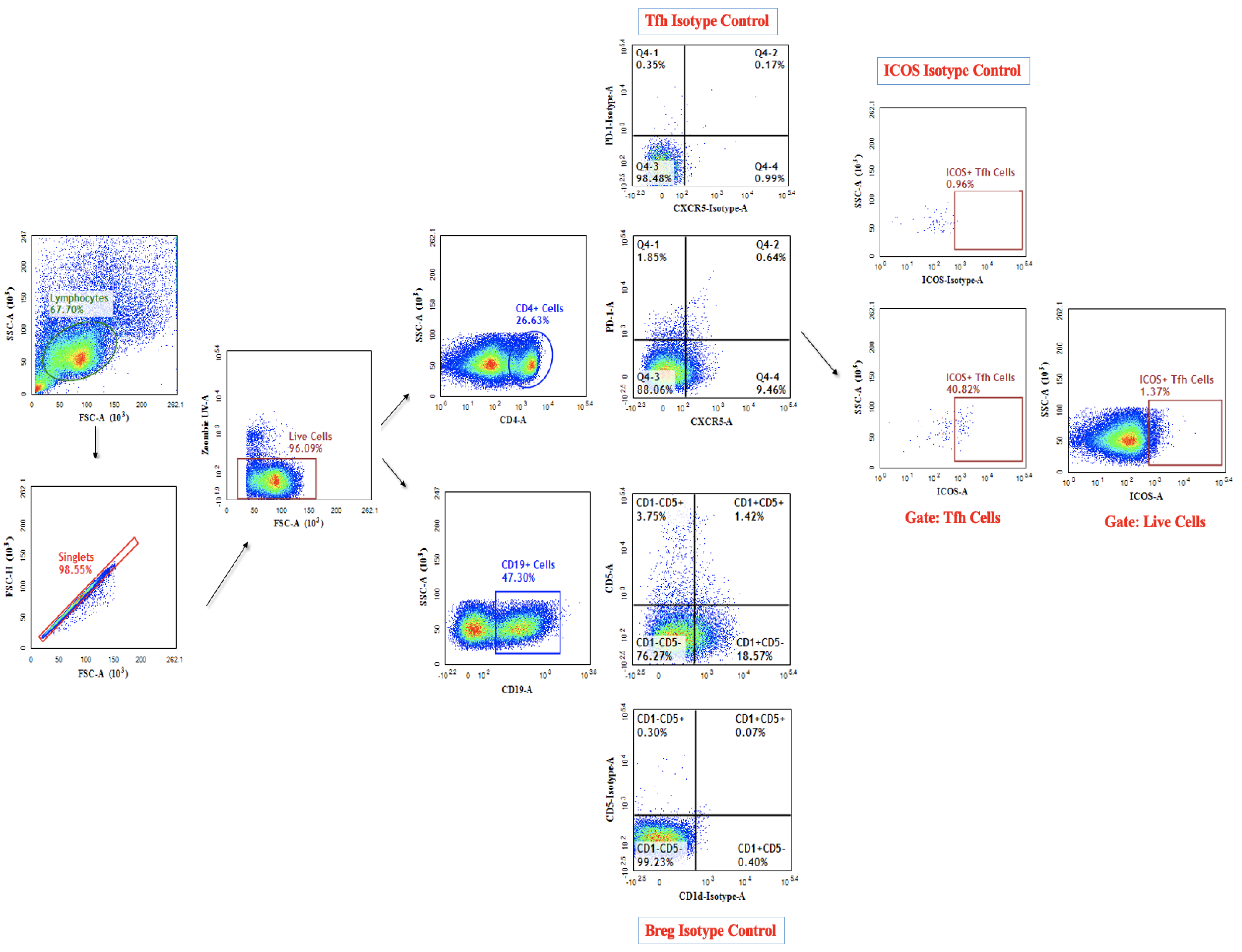

Supplement: Supplementary Figure S1 — Gating strategy used to define Tfh, ICOS+ Tfh, and Breg cells in the spleen of asthmatic mice. Within the live CD4+ T cells, Tfh cells were identified as CXCR5+PD-1+ cells. ICOS+ Tfh cells were identified from the Tfh cells as CXCR5+PD-1+ ICOS+ cells. Within the live CD19+ B cells, Breg cells were identified as CD5+CD1d+ cells. [file Image_1.tif]

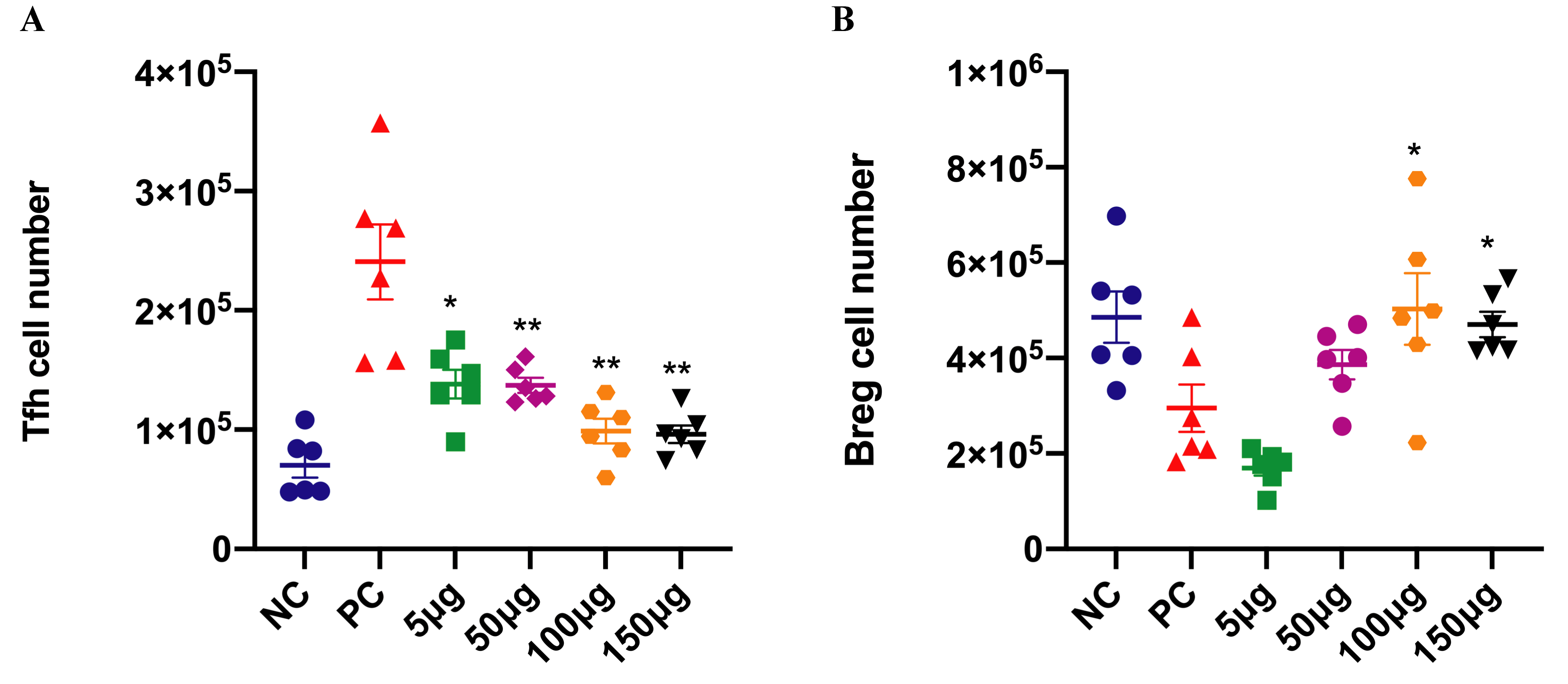

Supplement: Supplementary Figure S2 — Absolute number of Tfh and Breg cells in the spleen of asthmatic mice. (A) Tfh cell numbers in each group. (B) Breg cell numbers in each group. (n=6 per group). Values are means ± SEMs. *P < .05, **P < .01 compared to the PC group. NC, Negative group; PC, Positive group, 5 μg, 50 μg, 100 μg, 150 μg: 5 μg, 50 μg, 100 μg, 150 μg rAlt a 1 SCIT group. [file Image_2.tif]
