# Supplementary material for: Immunotherapy With Recombinant Alt a 1 Suppresses Allergic Asthma and Influences T Follicular Cells and Regulatory B Cells in Mice
Source: Front Immunol. 2021 Nov 5;12:747730. doi: 10.3389/fimmu.2021.747730 (PMC8602824; doi:10.3389/fimmu.2021.747730)
Supplement: Supplementary file 3 [file Table_1.docx]

| Group | Sensitization | Challenge | SCIT | Challenge |
| --- | --- | --- | --- | --- |
| NC | PBS | PBS | PBS | PBS |
| PC | 10μg *Alt* /1mg Alum | 25μg *Alt* | PBS | 25μg *Alt* |
| 5μg | 10μg *Alt* /1mg Alum | 25μg *Alt* | 5μg rAlt a 1 | 25μg *Alt* |
| 50μg | 10μg *Alt* /1mg Alum | 25μg *Alt* | 50μg rAlt a 1 | 25μg *Alt* |
| 100μg | 10μg *Alt* /1mg Alum | 25μg *Alt* | 100μg rAlt a 1 | 25μg *Alt* |
| 150μg | 10μg *Alt* /1mg Alum | 25μg *Alt* | 150μg rAlt a 1 | 25μg *Alt* |

Table S1 Outline of the SCIT protocol (n=6)

*Alt*: *Alternaria*, Alum: Aluminum. SCIT: subcutaneous immunotherapy. NC: Negative group, PC: Positive group, 5μg, 50μg, 100μg, 150μg: 5μg, 50μg, 100μg, 150μg rAlt a 1 SCIT group.
